# Hepatitis B and liver cancer knowledge and practices among healthcare and public health professionals in China: a cross-sectional study

**DOI:** 10.1186/1471-2458-10-98

**Published:** 2010-02-25

**Authors:** Jonathan Chao, Ellen T Chang, Samuel KS So

**Affiliations:** 1 Asian Liver Center at Stanford University, Stanford, California, USA; 2 Cancer Prevention Institute of California (formerly the Northern California Cancer Center), Fremont, California, USA

## Abstract

**Background:**

Chronic hepatitis B virus (HBV) infection is the leading cause of liver disease and liver cancer and a major source of health-related discrimination in China. To better target HBV detection and prevention programs, it is necessary to assess existing HBV knowledge, educational resources, reporting, and preventive practices, particularly among those health professionals who would be responsible for implementing such programs.

**Methods:**

At the China National Conference on the Prevention and Control of Viral Hepatitis on April 26-29, 2004, the Asian Liver Center at Stanford University partnered with the China Foundation for Hepatitis Prevention and Control to distribute a voluntary written questionnaire to Chinese healthcare and public health professionals from regional and provincial Chinese Centers for Disease Control and Prevention, health departments, and medical centers. Correct responses to survey questions were summed into a total knowledge score, and multivariate linear regression was used to compare differences in the score by participant characteristics.

**Results:**

Although the median score was 81% correct, knowledge about HBV was inadequate, even among such highly trained health professionals. Of the 250 participants who completed the survey, 34% did not know that chronic HBV infection is often asymptomatic and 29% did not know that chronic HBV infection confers a high risk of cirrhosis, liver cancer, and premature death. Furthermore, 34% failed to recognize all the modes of HBV transmission and 30% did not know the importance of the hepatitis B vaccine in preventing liver disease. Respondents who reported poorer preventive practices, such as not having personally been tested for HBV and not routinely disposing of used medical needles, scored significantly lower in HBV knowledge than those who reported sound preventive practices. Of note, 38% of respondents reported positive HBsAg results to patients' employers and 25% reported positive results to patients' schools, thereby subjecting those with positive results to potential discriminatory practices.

**Conclusions:**

These results indicate that there is a need for development of effective educational programs to improve HBV knowledge among health professionals and the general public to avoid missed vaccination opportunities, reduce misconceptions, and eliminate discrimination based on chronic hepatitis B in China.

## Background

The People's Republic of China carries the greatest burden of chronic hepatitis B virus (HBV) infection in the world, with a greater than 8% hepatitis B surface antigen (HBsAg) seroprevalence in a population of 1.3 billion people [1,2]. Individuals chronically infected with HBV have a one in four chance of dying from primary liver cancer or cirrhosis if not medically managed [3], and an estimated 60-80 percent of liver cancer cases in the Chinese population are caused by chronic HBV infection [4,5]. In comparison with HIV, which affects 700,000 Chinese [6], HBV has chronically infected an estimated 100 million Chinese, making it the most prevalent life-threatening infection in China [6]. Each year HBV kills 260,000 to 280,000 people in China [7]--more than tuberculosis (201,000) [8], HIV (39,000) [9], and malaria (<50) [10] combined.

As a blood-borne pathogen, HBV is primarily transmitted via perinatal/mother-to-child exposure, sexual intercourse, close household contact, needle-sharing, and occupational (health-care-related) exposure [11]. In China, where HBV infection is endemic, vertical transmission at childbirth is the predominant route of chronic infection [12]. Vertical transmission can be prevented through the administration of the hepatitis B vaccine and hepatitis B immune globulin at birth [13]. Among those who are chronically infected, routine screening for liver cancer can lead to early detection and a reduced risk of death [14].

Despite the presence of effective HBV and liver cancer screening measures and the implementation of a free hepatitis B vaccine program for all neonates by the Chinese government in 2002 [15], HBV continues to be a leading cause of mortality in China, with liver cancer ranking as the second most common cancer site among men and the fifth among women [16]. The persistence of high HBV-related mortality rates could be attributed in part to a lack of health provider knowledge, which can be a major obstacle to implementation of preventive programs. Yet, to our knowledge, the only study on health provider knowledge of HBV in China was a 1993 study by Clayton et al., who found poor HBV-related knowledge and widespread unsafe needle-use practices among village doctors in rural Minhou county in Fujian province [17]. With no wider studies on this topic, we set out to assess HBV-related knowledge among health professionals, including Chinese healthcare and public health professionals from regional and provincial Chinese CDC, health departments, and medical centers--i.e., those who should be the best-educated in viral hepatitis prevention. This survey enabled us to identify areas of HBV-related knowledge deficits, as well as associations of HBV preventive actions or demographics with levels of HBV knowledge. Our goal was to highlight the successes in HBV prevention while also determining the nature of obstacles in effective HBV prevention, so as to inform the design of future initiatives aimed at reducing the burden of HBV in China.

## Methods

### Study population

Voluntary written questionnaires were administered as part of the registration process at the China National Conference on the Prevention and Control of Viral Hepatitis held on April 26-29, 2004, in Hangzhou, China. The meeting was organized by the China Foundation for Hepatitis Prevention and Control, a national non-profit organization affiliated with the China CDC. Participants comprised public health and healthcare professionals from the provincial and regional CDC, health departments and medical centers. Conference attendees were asked to complete the questionnaire on-site at the conference at the time of registration, prior to being influenced by the conference proceedings. Signed consent to participate in the survey was obtained by the China Foundation for Hepatitis Prevention and Control. Among approximately 600 conference attendees, 250 completed the questionnaire; information was not obtained on attendees (including vendors) who did not complete the questionnaire.

### Questionnaire

The questionnaire was developed in-language (simplified Chinese) by the Asian Liver Center at Stanford University, based on its past experience with administering hepatitis B knowledge surveys in other populations, and with input from the China Foundation for Hepatitis Prevention and Control. The questionnaire consisted of four sections: i) HBV knowledge, ii) HBV prevention and reporting practices, iii) HBV educational resources, and iv) demographics and personal HBV-related health history. The knowledge section tested basic HBV and liver cancer knowledge concerning disease transmission, age-group risk, testing, incidence, whether treatment is curative, symptoms, outcomes, causes of liver cancer, prevention of perinatal transmission, modes of transmission, preventive measures, vaccination, and management of infected individuals. The section on HBV practices surveyed local test reporting, local restrictions on infected individuals, local vaccination practices, and needle disposal policies. The third section surveyed local HBV education. The fourth section surveyed participant demographics and personal HBV preventive actions.

### Statistical methods

An overall knowledge score was determined based on the sum of correct answers to the 16 knowledge-based questions. A correct response to each question received one point; incorrect and missing responses received no points. Knowledge questions that had multiple correct answers were awarded one point only if respondents selected all correct answer choices. Questions on local HBV practices, HBV education, and participant HBV preventive actions were categorized as "yes" if the participant reported performing the action and "no" if he or she did not. Missing responses were excluded because they were considered to be uninformative.

Univariate associations of demographic characteristics, participant HBV preventive practices, local HBV practices, and local HBV educational resources with knowledge score were assessed using linear regression to estimate the difference of total knowledge score (in continuous points with a corresponding 95% confidence interval [CI]) between groups. We grouped the 35 provinces and other administrative areas in China into 3 regions (East, Central, West) based on the regions defined in a 2004 study of hepatitis B vaccine coverage done by the Chinese Ministry of Health [18]. Covariates that were marginally (p < 0.10) associated with knowledge score were included in multivariate regression models for knowledge score. Multivariate models were adjusted for rural/urban status of residence, education level, occupation, whether or not the respondent had been tested for HBV, local test result notification practices, and local availability of free vaccines. Data were analyzed using SAS version 9.1.3 (SAS Institute, Cary, NC).

## Results

The demographic and some HBV-related characteristics of the study population are shown in Table 1. Most participants were male, urban, university-educated healthcare or public health professionals.

**Table 1:** Distribution of demographic characteristics and hepatitis B preventive practices among health professionals in China

Characteristic	*N*	(%)	Characteristic		*N*	(%)
**Sex**			**Education level**			
Male	146	60.1	Elementary		1	0.4
Female	97	39.9	Middle school		1	0.4
Unknown	6		High school		18	7.4
**Province of residence**			Vocational		29	11.8
Heilongjiang	4	1.7	University/college		181	73.9
Jilin	13	5.6	Graduate/professional degree		15	6.1
Liaoning	3	1.3	Unknown		4	
Beijing	16	6.9	**Occupation**			
Inner Mongolia	4	1.7	Physician		83	34.6
Gansu	26	11.2	Nurse or midwife		5	2.1
Xinjiang	2	0.9	Elementary school teacher/admin		1	0.4
Qinghai	10	4.3	Social worker		13	5.4
Ningxia	2	0.9	Government public health worker		42	17.5
Shanxi	7	3.0	Local public health worker		83	34.6
Hebei	18	7.8	Hospital administrator		13	5.4
Shandong	9	3.9	Unknown		9	
Jiangsu	14	6.0	**Family history of chronic hepatitis B**			
Henan	19	8.2	Yes		72	30.8
Shaanxi	20	8.6	No		162	69.2
Sichuan	5	2.2	Unknown		15	
Tibet	1	0.4	**Family history of liver cancer**			
Chongqing	2	0.9	Yes		19	8.5
Hubei	4	1.7	No		204	91.5
Anhui	3	1.3	Unknown		26	
Shanghai	3	1.3	**Ever been tested for HBV**			
Zhejiang	18	7.8	Yes		225	94.9
Jiangxi	4	1.7	No		12	5.1
Fujian	2	0.9	Unknown		12	
Hunan	2	0.9	**Vaccinated against HBV**			
Yunnan	2	0.9	Yes		195	81.6
Guangxi	5	2.2	No		44	18.4
Guangdong	14	6.0	Unknown		10	
Unknown	17		**Children vaccinated against HBV**			
**Rural/urban status of residence**			Yes		226	95.8
Rural	39	16.0	No		10	4.2
Urban	205	84.0	Unknown		13	
Unknown	5		**Ever heard of the Jade Ribbon Campaign**			
**Population of town/city of residence**			Yes		88	37.3
<10,000	17	7.0	No		148	62.7
10,000-50,000	75	30.9	Unknown		13	
500,000-1 million	42	17.3				
>1 million	109	44.9				
Unknown	6					

### Hepatitis B knowledge level

Out of a total knowledge score of 16, the median knowledge score was 13 (81%) correct (range = 4 to 16); 13 individuals (5%) responded correctly to all of the knowledge questions. Although five questions were correctly answered by more than 90% of participants, there was deficient HBV knowledge in several areas (Table 2). For example, 34% of respondents did not know that chronic HBV infection does not usually have symptoms and 29% did not know that chronic HBV infection can lead to liver cirrhosis, liver cancer, and premature death. Another 19% of respondents did not know that both HBV and hepatitis C virus (HCV) infection can lead to chronic (lifelong) infection. Thirty-four percent were unaware that contaminated blood, contaminated needles, unprotected sex with an infected person, and birth to an infected mother are all modes of HBV transmission. Furthermore, 30% of individuals did not know that giving the hepatitis B vaccine and avoiding the reuse of needles are two of the best ways to prevent HBV transmission. Only 31% of health workers knew that alpha-fetoprotein (AFP), alanine aminotransferase (ALT), and ultrasound tests should be performed to test liver function and screen for liver cancer in someone chronically infected with HBV.

**Table 2:** Distribution of hepatitis B and liver cancer knowledge and local hepatitis B practices among health professionals in China

Survey question		*N*	(%)
**HBV knowledge**			
*Which of the following viruses is transmitted like HIV?*			
Correct		228	91.6
Incorrect/missing		21	8.4
*Which of the following age groups, if infected, is at highest risk of developing chronic HBV infection?*			
Correct		223	89.6
Incorrect/missing		26	10.4
*Which of the following blood tests measures protection against HBV?*			
Correct		209	83.9
Incorrect/missing		40	16.1
*How many people in China have chronic HBV infection?*			
Correct		234	94.0
Incorrect/missing		15	6.0
*Which of the following is a curative treatment for HBV?*			
Correct		216	86.8
Incorrect/missing		33	13.2
*Does chronic hepatitis B usually cause symptoms?*			
Correct		165	66.3
Incorrect/missing		84	33.7
*What are the potential complications of chronic HBV infection?*			
Correct		177	71.1
Incorrect/missing		72	28.9
*What is the most common cause of liver cancer in China?*			
Correct		235	94.4
Incorrect/missing		14	5.6
*If a pregnant woman is an HBV carrier, which of the following is the best way to prevent perinatal transmission?*			
Correct		214	85.9
Incorrect/missing		35	14.1
*Which of the following viruses is transmitted by contaminated water?*			
Correct		240	96.4
Incorrect/missing		9	3.6
*Which of the following forms of viral hepatitis is vaccine-preventable?*			
Correct		239	96.0
Incorrect/missing		10	4.0
*Which of the following forms of viral hepatitis can lead to chronic hepatitis?*			
Correct		201	80.7
Incorrect/missing		48	19.3
*What are the modes of HBV transmission?*			
Correct		164	65.9
Incorrect/missing		85	34.1
*What are the best ways of preventing transmission of HBV?*			
Correct		175	70.3
Incorrect/missing		74	29.7
*Who should be vaccinated if they are not protected against HBV?*			
Correct		221	88.8
Incorrect/missing		28	11.2
*What are the tests used to monitor patients with chronic HBV infection?*			
Correct		77	30.9
Incorrect/missing		172	69.1
**HBV health practices**			
*If someone tests positive for HBV, do you report the results to the patient?*			
Yes		202	82.1
No		44	17.9
*If someone tests positive for HBV, do you report the results to the patient's employer?*			
Yes		93	37.8
No		153	62.2
*If someone tests positive for HBV, do you report the results to the patient's school?*			
Yes		61	24.8
No		185	75.2
*If someone tests positive for HBV, do you report the results to the health department?*			
Yes		105	42.7
No		141	57.3
*Does your city/village prohibit HBV-infected individuals from working for the government?*			
Yes		25	13.0
No		167	87.0
*Does your city/village prohibit HBV-infected individuals from attending schools?*			
Yes		53	27.6
No		139	72.4
*Does your city/village require HBV-infected individuals to have separate eating utensils/plates?*			
Yes		35	18.2
No		157	81.8
*Does your city/village prohibit HBV-infected individuals from working in healthcare?*			
Yes		52	27.1
No		140	72.9
*What population groups receive free hepatitis B vaccines in your city/village?*			
Vaccines are free for newborns (± other groups)		222	89.5
Vaccines are free for health workers only		5	2.0
Vaccines are only for purchase		19	7.7
Vaccines are not available		2	0.8
*What do clinics/hospitals do with used needles in your city/village?*			
Single use and destroy on site		186	75.3
Single use but collected by supplier for disposal		19	7.7
Disinfect and reuse		6	2.4
Don't know		36	14.6
*Do you display or have available HBV educational material in your workplace?*			
Yes, we have posters		69	27.7
Yes, printed information is available by request		62	24.9
Both		69	27.7
No		49	19.7

HBV: hepatitis B virus

### Hepatitis B prevention, reporting practices, and public educational resources

Of the individuals surveyed, 18% said that they do not report a positive HBsAg screening test result directly to the patient (Table 2). Conversely, 38% reported a positive HBsAg test result to the patient's employer and 25% reported a positive result to the patient's school--practices that exacerbate employer- and school-based discrimination against HBsAg-positive individuals in China [19,20]. Likewise, 28% of health workers reported that an infected individual in their city was prohibited from attending school and 27% said that infected individuals are prohibited from entering the healthcare industry. With regard to local vaccination policies, 90% of health workers reported that their city/village, at the minimum, provides free vaccines for newborns, and most do so for healthcare workers as well. However, 15% of health professionals did not know how their hospital or clinic handled used needles and 20% did not have health information about HBV available at their workplace.

### Hepatitis B preventive actions

Nearly all participants reported that they and their children had received the hepatitis B vaccine series and that they themselves had been tested for HBV infection (Table 1). Nearly one-third had a family history of chronic hepatitis B and 8.5% had a family history of liver cancer. Thirty-seven percent had heard of the Jade Ribbon Campaign, an international initiative launched by the Asian Liver Center at Stanford University in 2001 to increase hepatitis B and liver cancer awareness and prevention.

### Associations with hepatitis B knowledge level

After adjusting for other influences on HBV-related knowledge, individuals from rural provinces scored, on average, 1.0 point lower (95% CI: -1.6, -0.3 points) in HBV-related knowledge questions compared with those from urban provinces (Table 3). In addition, individuals who had completed only high school or vocational school scored 1.2 and 1.3 points lower, respectively, than individuals who had completed college or university. Of the occupations surveyed, nurse/midwives scored 2.4 points lower (95% CI: -4.3, -0.5) than physicians on HBV-related knowledge questions, while elementary school staff scored 3.9 points lower (95% CI: -7.4, -0.4). Health professionals who reported having personally been screened for HBV scored 1.2 points higher (95% CI: 0.1, 2.3) than health workers who did not report having been tested. Having been vaccinated or having had one's children vaccinated against HBV was not significantly associated with differences in knowledge score.

**Table 3:** Differences in hepatitis B knowledge scores among health professionals in China

Characteristic		Unadjusted difference	95% Confidence Interval	p-value
Sex				
Male		0.0	reference	
Female		0.2	(-0.4, 0.7)	0.57
Region of residence				
Eastern		0.0	reference	
Central		0.3	(-0.3, 1.0)	0.29
Western		-0.8	(-1.5, -0.2)	0.01
Rural/urban status of residence				
Rural		-1.8	(-2.4, -1.1)	<0.001
Urban		0.0	reference	
Population of town/city of residence				
<10,000		-1.5	(-2.5, -0.5)	0.005
10,000-500,000		-0.5	(-1.0, 0.1)	0.12
500,000-1 million		-0.2	(-0.9, 0.5)	0.57
>1 million		0.0	reference	
Education level				
Elementary		0.7	(-3.2, 4.7)	0.71
Middle school		-0.3	(-4.2, 3.7)	0.90
High school		-1.8	(-2.7, -0.8)	<0.001
Vocational		-1.3	(-2.1, -0.5)	0.002
University/college		0.0	reference	
Graduate/professional degree		-0.3	(-1.4, 0.7)	0.56
Occupation				
Physician		0.0	reference	
Nurse or midwife		-5.0	(-6.8, -3.2)	<0.001
Elementary school teacher/admin		-3.2	(-7.2, 0.7)	0.11
Social worker		-0.3	(-1.5, 0.9)	0.62
Government public health worker		-0.2	(-1.0, 0.5)	0.57
Local public health worker		-0.2	(-0.8, 0.4)	0.51
Hospital administrator		-0.4	(-1.6, 0.7)	0.45
Family history of chronic hepatitis B				
Yes		0.0	(-0.6, 0.6)	0.92
No		0.0	reference	
Family history of liver cancer				
Yes		-0.6	(-1.5, 0.4)	0.25
No		0.0	reference	
Ever been tested for HBV				
Yes		1.9	(-0.7, 3.0)	0.002
No		0.0	reference	
Vaccinated against HBV				
Yes		0.5	(-0.2, 1.1)	0.19
No		0.0	reference	
Children vaccinated against HBV				
Yes		1.1	(-0.2, 2.5)	0.09
No		0.0	reference	
Ever heard of the Jade Ribbon Campaign				
Yes		0.0	(-0.6, 0.5)	0.92
No		0.0	reference	
**Hepatitis B health practices**				
*If someone tests positive for HBV, do you report the results to the ...?*				
Only to the patient (± health department)		0.0	reference	
To the patient and the patient's school or employer		-0.5	(-1.1, 0.1)	0.08
Only to the patient's school or employer		-1.9	(-2.6, -1.2)	<0.001
*Does your city/village restrict HBV-infected individuals from working for the government?*				
Yes		0.7	(-0.2, 1.7)	0.11
No		0.0	reference	
*Does your city/village restrict HBV-infected individuals from attending schools?*				
Yes		0.5	(-0.2, 1.2)	0.19
No		0.0	reference	
*Does your city/village require HBV-infected individuals to have separate eating utensils/plates?*				
Yes		0.0	(-0.8, 0.8)	0.97
No		0.0	reference	
*Does your city/village restrict HBV-infected individuals from working in healthcare?*				
Yes		0.2	(-0.5, 0.9)	0.56
No		0.0	reference	
*What population groups receive free hepatitis B vaccines in your city/village?*				
Vaccines are free for newborns (± other groups)		0.0	reference	
Vaccines are free for health workers only		-1.6	(-3.4, 0.3)	0.10
Vaccines are only for purchase		0.3	(-0.7, 1.2)	0.61
Vaccines are not available		-3.5	(-6.4, -0.6)	0.02
*What do hospitals do with used needles in your city/village?*				
Single use and destroy on site		0.0	reference	
Single use but collected by supplier for disposal		0.9	(-0.1, 1.9)	0.07
Disinfect and reuse		-3.2	(-4.8, -1.5)	<0.001
Don't know		0.5	(-0.2, 1.2)	0.26
*What forms of HBV educational material do you utilize in your workplace?*				
Posters		0.3	(-0.5, 1.1)	0.42
Printed information		0.4	(-0.3, 1.2)	0.28
Posters and printed information		0.5	(-0.3, 1.3)	0.22
None		0.0	reference	

		Adjusted difference*	**95% Confidence Interval**	**p-value**

		0.0	reference	
		-0.1	(-0.5, 0.4)	0.84
		0.0	reference	
		0.0	(-0.6, 0.6)	0.99
		-0.3	(-0.9, 0.3)	0.28
		**-1.0**	**(-1.6, -0.3)**	**0.004**
		0.0	reference	
		-0.5	(-1.5, 0.5)	0.32
		-0.1	(-0.7, 0.4)	0.67
		-0.1	(-0.7, 0.5)	0.79
		0.0	reference	
		0.3	(-3.1, 3.8)	0.85
		-0.6	(-4.0, 2.9)	0.75
		**-1.2**	**(-2.1, -0.3)**	**0.008**
		**-1.3**	**(-2.0, -0.5)**	**<0.001**
		0.0	reference	
		0.1	(-0.9, 1.1)	0.84
		0.0	reference	
		**-2.4**	**(-4.3, -0.5)**	**0.01**
		**-3.9**	**(-7.4, -0.4)**	**0.03**
		0.0	(-1.1, 1.1)	0.99
		0.3	(-0.4, 1.0)	0.42
		-0.1	(-0.7, 0.5)	0.77
		-0.2	(-1.3, 0.9)	0.73
		0.0	(-0.5, 0.5)	0.93
		0.0	reference	
		0.0	(-0.9, 0.9)	0.96
		0.0	reference	
		**1.2**	**(0.1, 2.3)**	**0.03**
		0.0	reference	
		0.5	(-0.2, 1.1)	0.15
		0.0	reference	
		0.3	(-0.9, 1.6)	0.62
		0.0	reference	
		0.0	(-0.4, 0.5)	0.84
		0.0	reference	
		0.0	reference	
		-0.2	(-0.8, 0.3)	0.37
		**-1.5**	**(-2.1, -0.9)**	**<0.001**
		0.2	(-0.6, 1.1)	0.59
		0.0	reference	
		0.5	(-0.1, 1.2)	0.09
		0.0	reference	

*Adjusted for urban/rural status of residence, education level, occupation, HBV testing practices, HBV test notification practices, and availability of free vaccines

HBV: hepatitis B virus

In terms of local HBV-related practices, health professionals who reported positive HBsAg test results only to schools and/or employers scored significantly lower (-1.5 points, 95% CI: -2.1, -0.9) than health workers who reported results only to the patient (with or without reporting to the health department). In addition, individuals who reported that their local hospitals disinfected and reused needles scored significantly poorer (-2.0 points, 95% CI: -4.1, 0.0) than those whose hospitals destroyed needles on-site after a single use. However, health workers who reported employment, schooling, and/or community restrictions on persons with chronic HBV infection in their city/village did not score significantly lower than health workers who reported no such restrictions.

## Discussion

Our results show that even in our sample of highly educated healthcare providers and public health professionals, HBV- and liver-cancer-related knowledge, practices, and education were deficient, especially among health workers from rural provinces of China, those with lower general education, and nurses, midwives, and elementary school staff. HBV-related knowledge scores were higher among participants from areas with patient-directed HBV test notification procedures and strict needle disposal practices, suggesting that HBV knowledge and sound health policy are intertwined. Taken together, our findings demonstrate the need for improved, targeted education and training of health professionals in regions with low HBV-related knowledge in order to increase preventive practices and decrease rates of transmission and morbidity and mortality due to chronic HBV infection.

### Recent progress in China

Several noteworthy advances have been made in HBV prevention and knowledge in China during the past decade. In 1992, the Chinese government recommended routine hepatitis B vaccination of infants, but high vaccine prices and user fees barred all but the wealthiest persons from being vaccinated [15]. Therefore, in 2002, the Chinese Ministry of Health partnered with the GAVI Alliance (formerly the Global Alliance for Vaccines and Immunisation) to distribute hepatitis B vaccines to economically disadvantaged provinces and counties in western and central China. Furthermore, in 2005, all charges and user fees for the hepatitis B vaccine were abolished, making the vaccine free to all children in China. As a result of these programs, timely coverage with the birth dose of hepatitis B vaccine increased from 29.1% among children born in 1997 to 75.8% among children born in 2003; and completion of the hepatitis B vaccine series rose from 70.7% among children born in 1997 to 89.8% among those born in 2003. Coverage rates were even higher in the provinces and counties targeted by the China-GAVI project [15]. Additional gains in HBV-related knowledge and prevention among healthcare providers and public health professionals could spur further acceleration in the pace of HBV prevention and control throughout China, bringing vaccination rates ever closer to 100%--particularly in the underaddressed population of children and adolescents born before 2003. In this latter group, successful pilot catch-up vaccination programs [21-23], as well as strong evidence that such programs would be cost-saving in China [24], may provide the impetus needed to implement a nationwide universal catch-up vaccination program for children and adolescents.

### HBV knowledge

Perhaps due in part to the recent progress in HBV prevention in China, most HBV knowledge questions on our survey were correctly answered by more than half of the participants, and the median overall score was 81% correct. Nevertheless, we found that critical gaps in HBV knowledge are still present among trained health workers in China. In particular, one-third of health workers in China were unaware of the typically asymptomatic nature of chronic HBV infection, as well as the endpoints of the disease, such as end-stage liver cirrhosis, liver cancer and premature death. Health workers lacking such knowledge may overlook the importance of screening asymptomatic children and adults, and vaccinating those who are susceptible. These observations are consistent with our findings that health workers who scored significantly higher on HBV knowledge-based questions had also been screened and vaccinated for HBV themselves.

Some health workers also fell short when it came to knowledge about the basics of preventing the further spread of the disease. One-third of health workers surveyed were not sufficiently informed about all the modes of transmission and the best ways to prevent transmission, including vaccination and safe needle disposal practices. Such lack of knowledge among health workers could translate into similarly deficient knowledge in the general population, thereby contributing to insufficient preventive practices that enable the persistence of high rates of HBV infection.

Based on our findings, these HBV knowledge deficits were most substantial in rural regions of China. This disparity may be partially attributable to the reported difficulties in HBV training, vaccine transportation, vaccine storage, and vaccine costs in rural areas, where 21.5 million live below the official "absolute poverty" line [17,25]. Indeed, a quarter of surveyed individuals reported that there are no free hepatitis B vaccines available in their city/village. Yet, it should be noted that vaccine availability was not necessarily associated with better HBV knowledge. For instance, individuals from cities/villages that provided free vaccines to health workers scored marginally lower on HBV knowledge-based questions than those from cities/villages with only purchasable vaccines. The reason for this paradox is unclear and may be attributable to a lack of accompanying provider education even when the vaccine itself is available.

Health worker knowledge concerning the importance of newborn vaccination was high. In 2002, infant hepatitis B vaccination was added to China's National Immunization Program, and the China-GAVI Project was launched [15]. Consistent with these government-led initiatives, our results show that the great majority of health workers knew that newborns have the greatest risk of becoming chronic HBV carriers, and that a timely HBV birth dose given to newborns is the best way to prevent transmission.

Our results also showed that nurses, midwives, and elementary school staff had significantly lower HBV knowledge scores than physicians, albeit based on a small sample size. This result suggests the need to target HBV education to nurses and midwives, some of whom are the only health workers present at the time of newborn delivery in rural regions, where low-income families tend to give birth at home [15]; and to provide better HBV information to elementary school teachers and administrators, who can play a critical role in promoting catch-up hepatitis B vaccination among vulnerable schoolchildren who were not vaccinated at birth.

### Additional obstacles to prevention

While improving provider and health worker knowledge regarding HBV prevention is a necessary first step toward HBV prevention, other additional obstacles often hinder translation of knowledge into effective preventive practices. In 1993 Clayton et al. performed a study using a different self-administered questionnaire to evaluate HBV knowledge and preventive practices among 260 village doctors in the mostly rural Minhou county of the Fujian Province [17]. They found that provider knowledge often did not translate into preventive practices with their patients. According to Clayton et al., 90% local village doctors in Minhou county correctly identified the modes of HBV transmission. However, when asked to give medical advice to imaginary patients, only 8.5% of the survey respondents advised vaccination and only nine (3.5%) cautioned an HBsAg-positive patient against giving blood. The authors of the study suggested that HBV information may have been memorized, but not enough training was given on how to put that information into practice. Their findings contrast with those from a 2007 study done in the San Francisco Bay Area of California, where greater provider knowledge about the risk factors/guidelines of HBV-associated primary liver cancer in Asian Americans was correlated with greater HBV screening efforts [26]. This country-to-country difference suggests that gaps in implementation of preventive practices in China cannot be accounted for by lack of knowledge alone.

One explanation could be the stigma of chronic hepatitis B--a widespread basis for employment, school, and social discrimination in China, but not in the United States. A quarter of all health workers we surveyed stated that they report a positive test result to the patient's school, and an even greater proportion report the results to the patient's employer. These reporting policies suggest that an underlying prejudice against chronically infected individuals in China may deter at-risk individuals from getting screened (even if screening is available) and subsequently receiving the vaccine or being monitored for liver cancer. In an effort to curtail HBV-related discrimination, the Chinese Ministry of Health and Ministry of Human Resources and Social Security co-issued a notice in 2007 stating that an employer shall not refuse to employ or dismiss an HBV carrier because of his or her infection status, unless the person is banned from the job by laws, regulations, or Ministry of Health rules [27]. In December 2009, the Ministry of Health further announced that it would soon prohibit the long-standing practice of mandatory HBV testing during physical exams given for employment or school enrollment, signaling further progress in fighting discrimination [28]. However, methods for supervising and enforcing this new policy are unclear. Furthermore, poor HBV-related knowledge in the general population--and, most likely, among health professionals--still perpetuates social discrimination against chronically infected individuals in China [19,20].

### Study limitations

Our research should be viewed with the following limitations in mind. Firstly, all collected data were self-reported and therefore not verifiable. In particular, there was some inconsistency in the collected data (i.e., participants provided multiple answers to questions that asked for only one answer), indicating that some of the reported information was unreliable. Secondly, because our study was cross-sectional in nature, we cannot attribute causation to individual factors. (i.e., we could not show that being screened for HBV leads to greater HBV knowledge, or vice versa). Lastly, because of the non-random sampling of health workers, our conclusions have limited generalizability to other health workers in China, since all the participants were relatively well-educated health workers whose work specifically involved addressing health risks pertaining to liver cancer. However, given the inadequate HBV-related knowledge in this highly educated sample, it is reasonable to assume that other health workers have similar or poorer knowledge about HBV. Insofar as health providers are the individuals most trusted to provide health information and healthcare to the public at large, better education of healthcare providers and public health professionals is critical to disseminating HBV-related knowledge and preventive practices throughout the population of China.

### Implications for future initiatives

In spite of the limitations, our study also has several strengths. To our knowledge, there have been no other studies on HBV knowledge and practices performed on such a broad representation of health professionals from different provinces in China. Given the absence of prior data, our findings present novel and valuable information regarding the presence and nature of knowledge deficits among local health workers, as well as demonstrating a direct relationship between low HBV-related knowledge and insufficient HBV preventive practices.

## Conclusions

Our study highlights the continuing need for greater HBV knowledge in the rural reaches of China, as well as creative health strategies that address the complexities of translating HBV knowledge into effective prevention. In addition, our results emphasize the importance of establishing and enforcing patient privacy policies, as well as improving general awareness about HBV transmission, to abolish discrimination against chronically infected individuals. With the combined effort of HBV educated health workers, health providers, policy makers, scientists, and government officials, the People's Republic of China can make even greater strides toward eliminating HBV transmission and the burden of disease due to this lethal yet preventable infection.

## Competing interests

The authors declare that they have no competing interests.

## Authors' contributions

JC participated in data collection and interpretation and drafted the manuscript. ETC performed the statistical analysis, participated in data interpretation, and drafted and revised the manuscript. SKSS conceived of the study and its design and coordinated data collection. All authors participated in critically revising the manuscript, and read and approved the final manuscript.

## Pre-publication history

The pre-publication history for this paper can be accessed here:

http://www.biomedcentral.com/1471-2458/10/98/prepub
